# An Innovative Smartphone-Based Otorhinoendoscope and Its Application in Mobile Health and Teleotolaryngology

**DOI:** 10.2196/jmir.2959

**Published:** 2014-03-03

**Authors:** Cheng-Jung Wu, Sheng-Yu Wu, Po-Chun Chen, Yaoh-Shiang Lin

**Affiliations:** ^1^Department of Otolaryngology, Head And Neck SurgeryKaohsiung Veterans General HospitalKaohsiungTaiwan; ^2^Institute Of Clinical MedicineKaohsiung Medical UniversityKaohsiungTaiwan; ^3^Department of Otolaryngology, Head And Neck SurgeryTri-Service General Hospital, National Defense Medical CentreTaipeiTaiwan; ^4^Department of Industrial And Systems EngineeringChung Yuan Christian UniversityChungliTaiwan; ^5^Department of Radiation OncologyPingtung Christian HospitalPingtungTaiwan

**Keywords:** otorhinoendoscope, smartphone, mobile health, teleotolaryngology, telediagnosis

## Abstract

**Background:**

The traditional otorhinoendoscope is widely used in the diagnosis of a variety of ear and nose diseases, but only one doctor can use it at a time. It is also very difficult to share observations from one doctor with another doctor. With advances in electronic health technology, the extended potential application of smartphones to support medical practice or mobile health has grown steadily.

**Objective:**

The first phase of the study discussed how smartphones may be used for otorhinoscopic imaging and image management via an innovative adaptor. The second phase of the study was to evaluate the diagnostic capability of the smartphone-based otorhinoendoscope, as compared to the traditional otorhinoendoscope, and its application in mobile health and teleotolaryngology.

**Methods:**

We designed a unique adaptor to connect the otorhinoendoscope and smartphone in order to perform smartphone-based otorhinoendoscopy. The main aim was to transform the smartphone into an otorhinoendoscope. We devised a method that would allow us to use the smartphone’s camera to capture otorhinoscopic images. Using a freely available Web-based real-time communication application platform and the 3G (or WIFI) network, the smartphone-based otorhinoendoscope could synchronize the smartphone-based otorhinoscopic image with smartphones, tablet PCs, computer notebooks, or personal computers.

**Results:**

We investigated the feasibility of telemedicine using a smartphone, tablet PC, and computer notebook. Six types of clinical otorhinoscopic images were acquired via the smartphone-based otorhinoendoscope from six patients, which were examined in this study. Three teleconsultants (doctors A, B, and C) reviewed the six types of clinical otorhinoscopic images and made a telediagnosis. When compared to the face-to-face diagnosis, which was made in-person via a traditional otorhinoendoscope, the three teleconsultants obtained scores of a correct primary telediagnosis 83% (5/6), 100% (6/6), and 100% (6/6) of the time, respectively. When the clinical data were provided, the three teleconsultants obtained a correct secondary telediagnosis score of 100% (6/6), 100% (6/6), and 100% (6/6) of the time, respectively.

**Conclusions:**

The use of previously available technologies in the absence of any additional expensive devices could significantly increase the quality of diagnostics while lowering extraneous costs. Furthermore, this could also increase the connectivity between most isolated family doctors and remote referral centers.

## Introduction

Electronic health is defined as the use of information and communication technologies in support of health and health-related fields, including health care services, health surveillance, health literature, and health education, knowledge, and research [1]. Mobile health is a term used for the practice of medicine and public health and is supported by mobile devices, such as mobile phones, smartphones, tablet computers, and PDAs (personal digital assistants). Mobile health has emerged as a subsegment of electronic health [2]. Mobile health is now pushing the limits of how we acquire, transport, store, process, and secure raw and processed data to deliver meaningful results. Mobile health also supports health care workers who perform clinician-like duties where there are no doctors and can help to keep track of patients, which may not have been possible in the past [1,2]. There have been considerable advances in the field of mobile phone technology within the past few years. It is predicted that by 2017 there will be more mobile phones than people in the world. Currently, three-quarters of the world’s population have access to a mobile phone [3,4]. With nearly 5 billion mobile phone users worldwide, researchers are now realizing the great potential of using mobile health technology, such as smartphones, for electronic health services. Mobile health can also support the daily practice of health care workers via the dissemination of clinical updates, learning materials, and reminders [5], particularly in underserved rural locations, as well as in low-income and middle-income countries. With the development of smartphone technology, mobile health can support daily practice in the field of telemedicine.

Telemedicine is a technique that enables virtual online communication in real time among health care providers who are located in distant places and for patients in rural areas. There are several ways in which to perform telemedicine. In its most basic form, telemedicine is similar to a videophone, consisting of a camera, a monitor, and a microphone to transmit video and audio from one location to specialists in another location [6]. This enables the primary care doctor who is located in a rural area to discuss patients in detail with a specialist at another remote site in order to provide diagnostic and therapeutic advice. According to a World Health Organization (WHO) report, telemedicine is particularly useful in rural areas or in developing countries where access is limited to specialists and subspecialists. According to Vera-Domínguez J et al (2010), the use of telemedicine by a trained technician at the site where the patient is can provide information to the physician who specializes in this problem—this may be a referral hospital in another distant location [6]. Teleotolaryngology is defined as telemedicine in the specialty of otolaryngology and head and neck surgery. With advances in technology, the use of telemedicine to treat diseases has grown significantly. Diseases of the ear, throat, and nose are among the most common diseases, which can be diagnosed and treated via teleotolaryngology.

According to a WHO global report on chronic disease prevention, chronic otitis media is one of the major chronic diseases in low-income and middle-income countries [7]. When categorized by cause, global and regional projections of mortality and burden of disease for the years 2000, 2010, and 2030 indicated that hearing loss is projected to be among the top ten causes of burden of disease in high-income and middle-income countries according to the WHO report [8]. To address the widespread demand for raising awareness regarding ear and hearing diseases in global health, and thereby support international health policy and priority setting, we have devised an innovative device to help diagnose chronic ear disease in the field of telemedicine.

**Figure 1:**
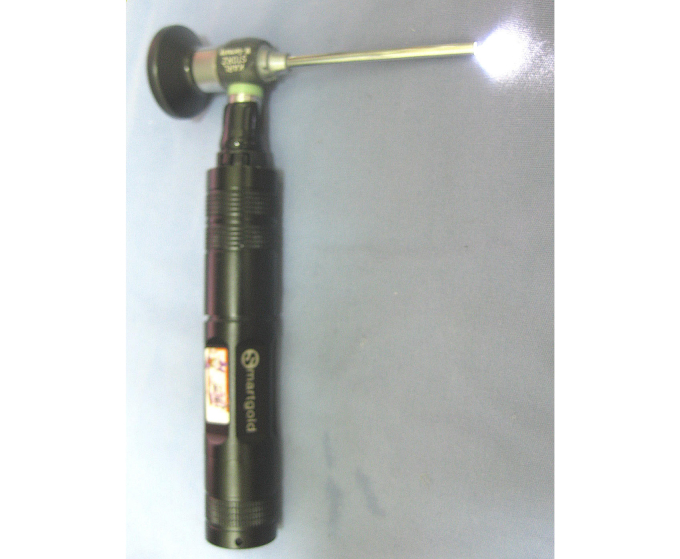
A traditional commercial otorhinoendoscope is widely used to help diagnose a variety of ear and nose diseases, but it can only be used by one doctor at a time.

Our study was motivated by several reports on health care technology published by Rubisky et al (2008) and the WHO [9-11]. According to WHO reports, the majority of the world’s population does not have access to health care technologies [10,11]. For example, traditional otorhinoendoscopes (Figure 1) are widely used in the diagnosis of a variety of ear and nose diseases, but can be used by only one doctor at a time. In addition, it is also notably difficult for a doctor to share his or her observations with another doctor during an examination. This difficulty is particularly true for a junior primary care doctor, where it is highly difficult to precisely describe the eardrum and external auditory canal condition to someone during an examination. A traditional otolaryngological video endoscope system can be used as an alternative tool in the field of teaching and telemedicine. However, an affordable and reliable otolaryngological video endoscope system is also lacking in many developing countries due to its high cost, maintenance difficulty, and weight. Moreover, despite the availability of an otolaryngological video endoscope system, these systems were often of an inadequate type, non-functional, or handled incorrectly due to the lack of well-trained doctors. According to WHO reports, more than 50% of medical equipment is not being used in the developing world because it is too sophisticated or is in disrepair, or due to the lack of trained health workers and maintenance. On the other hand, the lower cost of mobile phones (smartphones) and their great mobility has attracted more people who can afford and make use of mobile technology, even in developing countries [12]. With advances in electronic health technology, the extended application of the smartphone in medicine has evolved over time. This article introduces an innovative device and discusses how smartphones can be used for otorhinoscopic imaging and image management, as well as its application in mobile health and teleotolaryngology.

## Methods

### Designing a Specific Adapter Device

**Figure 2:**
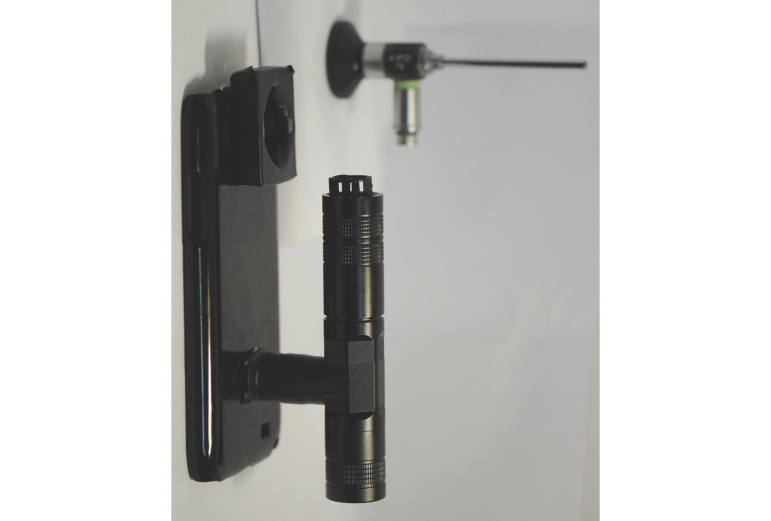
Self-designed specific adapter with portable light source.

**Figure 3:**
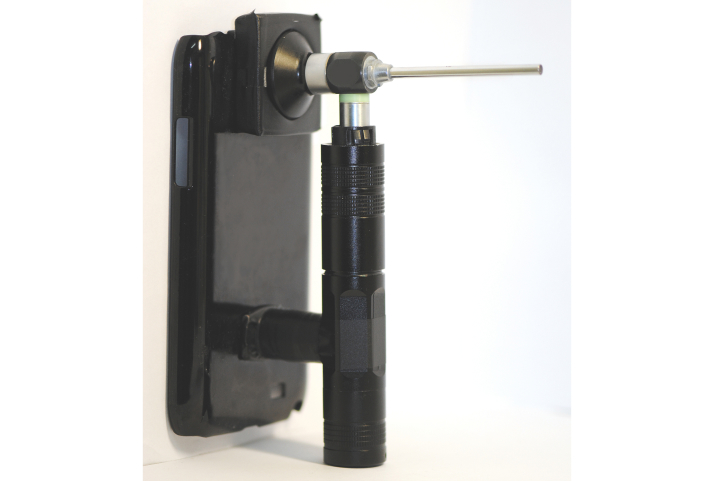
Prototype of a self-designed smartphone-based otorhinoendoscope.

The key concept of the smartphone-based otorhinoendoscope is to make a connection between the smartphone’s camera and the otorhinoendoscope. The process sounds simple, but several challenges had to be overcome. The aim was to transform a smartphone into an otorhinoendoscope. In developing our mobile health technology, the technical part of this study was challenging due to the lack of a commercial adapter to connect the smartphone and otorhinoendoscope. The challenge of our study was the need to create a specific adapter and portable light source. First, we designed an adapter to align the optical access of the otorhinoendoscope with the camera of the smartphone, which was employed to capture the otorhinoscopic images. For the smartphone-based otorhinoendoscopy, we designed a unique adapter (Figure 2) to connect the otorhinoendoscope to the smartphone. Figure 3 shows the connection of the otorhinoendoscope and specific adapter, which we refer to as the smartphone-based otorhinoendoscope. However, the smartphone-based otorhinoendoscope will not work without a portable light source. As a result, we designed a portable light source, which was modified from a commercial electric Light-Emitting Diode (LED) flashlight. The external LED light source of the device was powered by rechargeable batteries. The complete unit was extremely portable and easy to manipulate. No additional power supply unit or electrical sockets were required.

### Evaluation of Diagnostic Quality and Application in Telemedicine

**Figure 4:**
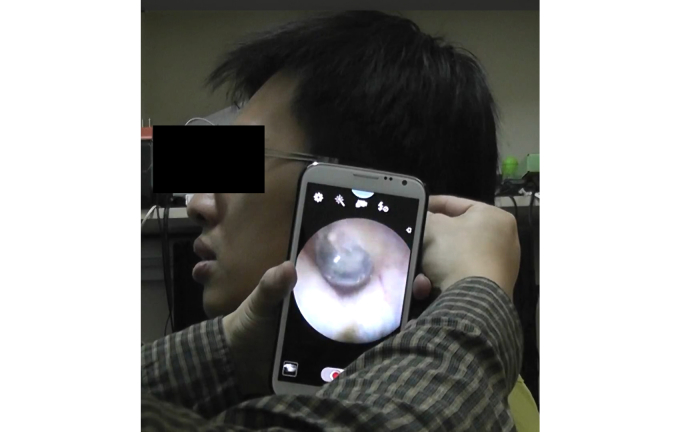
Using a smartphone-based otorhinoendoscope to evaluate an ear condition in a young male patient.

**Figure 5:**
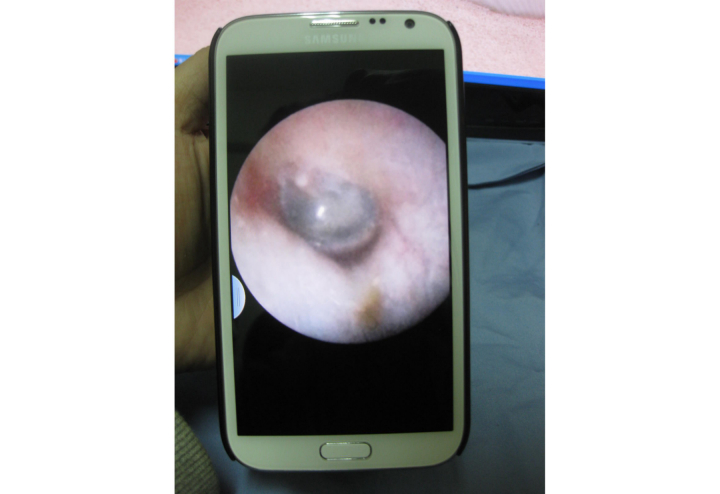
Clinical picture captured using a smartphone-based otorhinoendoscope.

Six patients (#1, 2, 3, 4, 5, and 6) were tested during a humanity mission in the mountainous area of Taiwan. Only patients who agreed to be evaluated using the traditional otorhinoendoscope and smartphone-based otorhinoendoscope were enrolled in this study. Written informed consent for participation in the project was obtained from the parents of all infants, and all procedures were approved by the Research Ethics Committee of the Kaohsiung Veterans General Hospital. Three teleconsultants who were professional otolaryngologists from a tertiary medical center in Taiwan participated. In this study, all otorhinoscopic images (Figures 4 and 5) were captured using a smartphone (Samsung, Galaxy Note II) with a built-in 8-megapixel camera with autofocus, macro mode, and zoom functions. A freely available Web-based real-time communication application platform (Google Plus Hangouts) was used to transmit the otorhinoscopic images via a third-generation network. An Internet connection was made available for the three teleconsultants. Two of the teleconsultants used a computer notebook with a 14-inch LCD monitor (Sony, VAIO and Acer, V5 Ultrabook) and the other teleconsultant used a tablet PC with a 10-inch LCD monitor (Apple, third-generation iPad).

Six types of clinical otorhinoscopic images were acquired from the six patients (#1, 2, 3, 4, 5, and 6) at the Kaohsiung Veterans General Hospital. The same otolaryngologist performed the face-to-face diagnosis, using a traditional otorhinoendoscope. After the face-to-face diagnosis was performed at a remote site, the raw data without details about the related clinical conditions were transmitted through the 3G network to a teleconsultant for a primary telediagnosis. Three teleconsultants—doctors A, B, and C—first reviewed the clinical images and reported their primary telediagnoses. All teleconsultants reviewed the cases independently from each other and directly responded with their primary telediagnoses using the real-time communication platform.

After the first telediagnosis was made, the patient’s clinical conditions were provided to each of the teleconsultants. Subsequently, a second telediagnosis was made. The three teleconsultants were asked to provide a specific telediagnosis (eg, acute otitis media, eardrum perforation) for each case in the primary and second telediagnoses, and only one telediagnosis was accepted. The telediagnoses were compared to the face-to-face diagnoses. The face-to-face diagnosis was assumed to be the correct diagnosis. Diagnostic agreement was defined as the concurrence between the telediagnoses and face-to-face diagnoses. The three teleconsultants were also asked to judge the quality of each image using the following scale: poor, fair, and good. The image quality was judged by the three teleconsultants subjectively. One of the limitations of this study worth noting is that the sample size was not large enough. In order to effectively develop this tool for clinical use, additional studies performed on a larger series of cases are required to confirm the effect of image quality on mobile health telediagnoses. Further larger series studies will be performed in the near future.

## Results

The clinical otorhinoscopic images acquired under the six conditions using the smartphone-based otorhinoendoscope were examined in this study (Table 1). Three teleconsultants reviewed the six conditions and performed the telediagnoses. The same otolaryngologist determined the face-to-face diagnosis for each case using the traditional otorhinoendoscope. The face-to-face diagnosis was assumed to be the correct diagnosis. Compared with the face-to-face diagnoses, the primary telediagnoses made by the three teleconsultants were 83% (5/6), 100% (6/6), and 100% (6/6) correct. After the patient’s clinical information was provided, the second telediagnoses made by the three teleconsultants became 100% (6/6), 100% (6/6), and 100% (6/6) correct. In one case, an atrophic scar of the tympanic membrane was overdiagnosed as an eardrum perforation due to poor image quality. Poor image quality was defined as severe loss of sharpness or color accuracy and with artifacts (distortion). Fair image quality was defined as mild loss of sharpness or color accuracy and without artifacts. Good image quality was defined as no loss of sharpness or color accuracy and without artifacts. Sharpness is affected by the lens of the smartphone (manufacturing quality, focal length). In this study, loss of sharpness was affected by camera shake due to the device being too light to hold steadily. It is worth noting that digital images from many smartphone cameras will sometimes lose the accuracy in color saturation due to the basic physical limitation of the lens. Further, a smartphone’s digital camera lens converts light into electricity and then that electricity is converted into pixels. The ability of the smartphone’s sensor chips to handle these conversions is not as good as a traditional digital camera. As such, the digital noise is higher in smartphones compared to traditional digital cameras. In our study, the quality of the images was judged as poor for 1 case (17%), fair for 2 cases (33%), and good for 4 cases (50%).

**Table 1:** Face-to-face diagnosis and telediagnosis of teleconsultants A, B, and C.

Patient	Face-to-face diagnosis	Primary telediagnosis^f^			Secondary telediagnosis^g^			Image quality of pictures		
		A^a^	B^b^	C^c^	A^a^	B^b^	C^c^	A^a^	B^b^	C^c^
#1	Allergic rhinitis	1^d^	1	1	1	1	1	Good	Good	Good
#2	Nasal polyp	1	1	1	1	1	1	Good	Good	Good
#3	Nasopharynx tumor	1	1	1	1	1	1	Fair	Fair	Fair
#4	Atrophic scar of the tympanic membrane	0^e^	1	1	1	1	1	Poor	Poor	Poor
#5	Acute otitis media	1	1	1	1	1	1	Fair	Fair	Fair
#6	Eardrum perforation	1	1	1	1	1	1	Good	Good	Good
Diagnostic agreement, n/n (%)		5/6 (83%)	6/6 (100%)	6/6 (100%)	6/6 (100%)	6/6 (100%)	6/6 (100%)			

^a^A: teleconsultant A

^b^B: teleconsultant B

^c^C: teleconsultant C

^d^1: agreement with face-to-face diagnosis

^e^0: disagreement with face-to-face diagnosis

^f^Primary telediagnosis: telediagnosis without clinical information

^g^Secondary telediagnosis: telediagnosis with clinical information

## Discussion

### Principal Findings

Our study examined the feasibility of the use of teleconsultation for ear and nose lesions using a smartphone-based otorhinoendoscope. The clinical pictures that were captured by using the newest generation of smartphones were excellent. In fact, the high resolution of the built-in autofocus camera in the new generation of smartphones resolves the problems of image quality found in previous reports [13,14]. These devices produced a *full* 1080 pixel *HD video image.* However, the images captured using the smartphone were not perfect—approximately 17% of the images were judged as being of poor quality by our teleconsultants. Poor image quality was defined as low sharpness, out-of-focus, inaccurate color, and with artifacts. It is important to remember that smartphones are phones first, then computers and also cameras. Unlike cameras, smartphones don’t have good anti-shake features. Also, the weight of device is light, making it difficult to hold steady. Although the image quality was not perfect, the three teleconsultants still obtained a 100% correct telediagnosis after the patient’s clinical information was provided. However, further studies performed on a larger series of cases are required to confirm the effect of image quality on mobile health telediagnoses.

It must be emphasized that the clinical information was not initially provided to the teleconsultants. To test the device’s real capability to formulate a telediagnosis, only the clinical images were initially provided. One of our patients had an atrophic scar of the eardrum. She had a history of traumatic eardrum perforation. An atrophic scar of the eardrum can be easily diagnosed using a pneumatic otoscope and clinical information. Without the most important clinical information and related history, one of our teleconsultants overdiagnosed at the primary telediagnosis. This result may be explained by the fact that in some cases, using the image alone is not sufficient to achieve a correct diagnosis. The correction rate of the telediagnosis was not attributed to a single factor. The quality of the telediagnosis was determined using the quality of the clinical image, the accompanying clinical information, and the experience of the teleconsultant.

A number of common treatable diseases account for the vast majority of ear and nose disease burdens in developing countries, such as chronic otitis media, allergic rhinitis, acute otitis media, and hearing impairment. There is a high demand for otolaryngologists in these developing countries. Most of these ear and nose diseases are readily treatable with the consultation of an otolaryngology specialist. With the help of mobile health technology, most otolaryngological diseases can be managed at a local health unit after a telediagnosis has been made by the teleconsultants. According to Haegen TW’s report (2004), teleotolaryngology resulted in the avoidance of 22.7% of consults. Only 13.0% of patients required traditional face-to-face otolaryngology consultation. These studies demonstrated the ability of teleotolaryngology to function in a general otolaryngological setting. For teleotolaryngology, suitable equipment is required to properly transmit audio-visual data so that otolaryngologist specialists and audiologists can benefit from the data in their examination and treatment of the patient. In addition, the application of teleotolaryngology can help patients to minimize their treatment expenses and to evaluate their treatment in a more efficient manner [15,16]. The possible contributions of mobile health can result in a significant health gain for both the individuals and communities in developing countries.

New acute otitis media treatment guidelines emphasize awareness. The American Academy of Pediatrics has released the new 2013 guidelines for the management of acute otitis media, which provide more strict criteria to limit unnecessary antibiotic prescriptions, and advice against prophylactic antibiotic treatment for children with recurrent infections [17]. Approximately 70% of children who present with ear infections get better on their own within three days and 80% are better within a week according to the new guidelines [17]. Overtreatment of ear infections with antibiotics in preschoolers may cause antibiotic resistance and has caused millions of unnecessary visits and prescriptions for antibiotics in the United States. Currently, physicians diagnose ear infections using otorhinoendoscopes to examine the eardrum. With advances in mobile health technology, primary care would able to take pictures via the smartphone-based otorhinoendoscope, and telediagnosis could be made by sending the pictures digitally to a remote otolaryngologist. The smartphone-based otorhinoendoscope is very easy to operate. Even parents could potentially record a clinical image without a doctor’s help. It is impossible for a patient to go to the outpatient clinic for an otological examination by an otolaryngologist daily to see if their eardrum condition has improved. However, it is possible for parents to take clinical images using these devices at home daily. The smartphone-based otorhinoendoscope has the potential to change the doctor’s practice patterns of overutilizing antibiotics for ear infections. Currently, otolaryngologists can wait to see if a child’s infection improves or if antibiotic treatment is warranted after the series of clinical images are obtained from parents.

**Figure 6:**
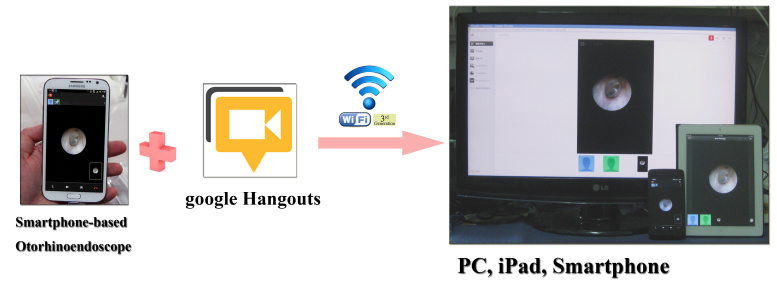
The smartphone-based otorhinoendoscope has a significant advantage in transmitting images to a smartphone, notebook, tablet PC, or personal computer via a freely available Web-based real-time communication application platform (Google Plus Hangouts in the Android system).

Technologies designed for developed countries are often incompatible with a developing country’s infrastructure, habits, and culture. Thus, local users must develop their own electronic health technology [4,5]. The development of a smartphone-based otorhinoendoscope may solve this problem. The complete unit of our smartphone-based otorhinoendoscope is extremely portable and easy to maneuver. No additional power supply unit or electrical sockets are needed, which is very convenient in developing countries where a power supply may be lacking. The device can be taken to the patient for use instead of bringing the patient to the device for their examination. One of the most impressive elements of the device was that it could be used for medical applications in the field of telemedicine. Using a network connection, the device could serve as a good diagnostic tool for doctors in remote areas around the globe. The device delivers a higher level of magnification as compared with traditional otorhinoendoscopes, making it easier to visualize the auditory canal and tympanic membrane. The device also has a significant advantage in transmitting images to another smartphone, notebook, tablet PC, or personal computer (Figure 6) via a freely available Web-based real-time communication application platform (FaceTime in iOS system or Google Plus Hangouts in the Android system), providing an improved perspective for enhanced learning and decision-making. It can be used in a daily outpatient office practice to demonstrate the clinical signs of ear and nose diseases to the patient or even to teach students and younger residents. Even patients or parents themselves could monitor changes in the ear condition and capture an image of the lesion using these devices and send the images digitally. The use of current widely available mobile technologies [18], in the absence of any additional otolaryngological video endoscope system, could significantly increase quality diagnostics and lower extra costs.

The cost of our specific adapter device was less than US$350. In our research, we used a refurbished smartphone, which was about US$300. A portable light source, which was modified from a commercial electric LED flashlight, was roughly US$40 and the adapter case cost about US$10. With little additional equipment required, the proposed device is a cost-effective smartphone-based otorhinoendoscope.

### Conclusions

Medical facilities in developing countries and rural regions are lacking. Advanced diagnostic tools are quite rare and very often there are no subspecialists providing services to these remote areas. In a surprising twist on technology, mobile phone use in developing countries has surpassed that of developed areas according to a recent World Bank report. There is also increasing connectivity between most isolated doctors and distant referral centers or hospitals. Thus, a smartphone-based otorhinoendoscope has the potential to become an easily applicable tool for everyone and a new approach to enhanced self-monitoring of ear and nose diseases, particularly in developing countries.

The present work is the first study on mobile health in the field of telemedicine using smartphones in the otolaryngological field. More prospective, randomized clinical studies are needed to test the application of the smartphone-based otorhinoendoscope in the field of mobile health and teleotolaryngology.

## Abbreviations

LED: Light-Emitting Diode
PDA: personal digital assistants
WHO: World Health Organization
